# Dexmedetomidine Versus Dexamethasone as Adjuvants to Bupivacaine for Wound Infiltration After Caesarean Section Under Spinal Anaesthesia

**DOI:** 10.7759/cureus.116221

**Published:** 2026-09-14

**Authors:** Shabnam Goyal, Pooja Gunwal, Deepak K Saini, Samriti Manhas, Parul Mullick

**Affiliations:** 1 Department of Anaesthesiology, Vardhman Mahavir Medical College & Safdarjung Hospital, New Delhi, IND; 2 Department of Anaesthesiology, Maharishi Chyawan Government Medical College and Rao Tularam Hospital, Koriawas, IND; 3 Department of Orthopaedics, Maharishi Chyawan Government Medical College and Rao Tularam Hospital, Koriawas, IND; 4 Department of Orthopaedics, Vardhman Mahavir Medical College & Safdarjung Hospital, New Delhi, IND

**Keywords:** bupivacaine, caesarean section, dexamethasone, dexmedetomidine, spinal anaesthesia, wound infiltration

## Abstract

Objective: Postoperative pain control is central to recovery after caesarean section. As wound infiltration is a standard multimodal analgesia component, we compared dexmedetomidine and dexamethasone as bupivacaine adjuvants for wound infiltration in women undergoing elective caesarean section under spinal anaesthesia, focusing on analgesic performance and safety.

Methods: This prospective, randomised, double-blind trial enrolled 90 American Society of Anesthesiologists (ASA) physical status II women scheduled for elective caesarean section, divided equally into three groups: 0.25% bupivacaine alone (Group B), bupivacaine with dexmedetomidine 1 μg/kg (Group BDM), or bupivacaine with dexamethasone 8 mg (Group BDX). The main measure was total tramadol use over the first postoperative day; other measures included time to first rescue dose, Visual Analogue Scale (VAS) pain scores, patient satisfaction, haemodynamic readings, sedation, and adverse events.

Results: Tramadol use varied significantly by group (P<0.0001) - lowest in Group BDM (87.33 ± 17.40 mg), followed by Group BDX (129.43 ± 15.61 mg) and Group B (150.33 ± 14.59 mg). Time to first rescue analgesia was longest in Group BDM (6.00 ± 0.10 hours), then Group BDX (4.00 ± 2.03 hours) and Group B (1.87 ± 0.35 hours) (P<0.0001). Both adjuvants outperformed bupivacaine alone for pain control, with dexmedetomidine giving the strongest analgesia and highest satisfaction scores. Haemodynamics, sedation, and adverse event rates were similar across groups.

Conclusion: Adding dexmedetomidine or dexamethasone to bupivacaine improved postoperative analgesia after caesarean section. Dexmedetomidine provided greater opioid sparing, longer analgesia, superior pain control, and higher patient satisfaction without compromising safety, supporting its use as an effective adjunct to bupivacaine wound infiltration.

## Introduction

Rates of caesarean section have been climbing steadily around the world, a trend tied to changes in obstetric care and wider access to hospital-based delivery services, which has pushed recovery-focused care higher up the priority list for obstetric anaesthetists. Good pain control after surgery does more than keep a woman comfortable. It helps her get up and move sooner, care for and feed her newborn, and avoid the downsides that come with heavy opioid use. This is why current anaesthesia practice leans towards multimodal analgesia strategies that provide effective pain relief while keeping opioid use to a minimum [1-5].

Of the analgesic options on offer, infiltrating the incision itself with a local anaesthetic stands out for being simple to perform, cheap, and easy to fold into a routine caesarean list. It works by dampening pain signals right where the tissue damage occurred, which fits neatly alongside the other elements of a multimodal plan. The catch is that local anaesthetics on their own don’t last especially long, and that limitation is what has driven researchers to look for additive agents that can stretch out the pain relief without adding to the complication rate [5-8].

Bupivacaine tends to be the local anaesthetic of choice for wound infiltration, thanks to its comparatively long-lasting effect and solid safety track record. A number of additives have been trialled alongside it in an effort to squeeze out more postoperative benefits. Dexmedetomidine works mainly by activating α₂-adrenoceptors, giving it its analgesic edge, while dexamethasone is thought to stretch out pain relief by damping down the inflammatory response triggered by surgical trauma. Earlier work has linked both drugs to lower analgesic use and longer-lasting pain control when paired with local anaesthetics [9-13]. Even so, head-to-head data comparing these two specific adjuvants in caesarean patients is thin on the ground, leaving it unclear which one actually performs better in practice [14, 15].

Enhanced Recovery After Surgery (ERAS) protocols push for opioid-sparing analgesia specifically to help women get back on their feet sooner after a caesarean. Pinpointing which adjuvant gives the better pain relief without sacrificing safety could therefore genuinely change clinical practice [2, 3]. Given how little direct comparison data exists, we designed this prospective, randomised, double-blind trial to weigh dexmedetomidine against dexamethasone as adjuvants to 0.25% bupivacaine for wound infiltration after elective caesarean section under spinal anaesthesia. Our main goal was to compare how much tramadol each group needed over the first 24 hours after surgery; alongside that, we looked at when patients first needed rescue analgesia, how their pain scores tracked, how satisfied they were, their haemodynamic response, and any treatment-related side effects.

## Materials and methods

This single-centre trial was led by the investigators themselves across the departments of Anaesthesiology and Obstetrics & Gynaecology of Vardhman Mahavir Medical College & Safdarjung Hospital, a tertiary teaching hospital in New Delhi, India, following a prospective, randomised, double-blind design. Before a single patient was enrolled, the protocol had already cleared the Institutional Ethics Committee and been logged with the Clinical Trials Registry-India (registration number: CTRI/2019/12/022513). Throughout, the study kept to the ethical standards laid out in the Declaration of Helsinki and to Good Clinical Practice requirements, and every participant signed a written consent form before joining.

Sample size was calculated based on a study by Abd El-Hamid et al. [16], in which mean total analgesic (morphine) consumption was 9.3 ± 1.6 mg in the bupivacaine group and 3.9 ± 1.4 mg in the dexmedetomidine plus bupivacaine group, and the proportion of patients requiring analgesia was 100% in the bupivacaine group versus 53.3% in the dexmedetomidine plus bupivacaine group. Taking these values as reference, the minimum required sample size with 99% power and a 1% level of significance was 28 patients per group. To reduce the margin of error, the total sample size was set at 90 (30 patients per group).

We screened women between 18 and 45 years old who were carrying a single baby at 37 weeks’ gestation or beyond and were booked for an elective lower-segment caesarean under spinal anaesthesia. To be included, a woman had to fall into American Society of Anesthesiologists (ASA) physical status II [17], be able to follow and use the Visual Analogue Scale (VAS) score [18], and be willing to sign the consent form.

Women were kept out of the study if they declined to take part, were sensitive to any of the trial drugs, could not safely have neuraxial anaesthesia, had pregnancy-induced hypertension or severe preeclampsia/eclampsia, carried a significant cardiac, respiratory, hepatic, renal, neurological, or psychiatric condition, needed regular opioids or analgesics for chronic pain, had an infection where the wound infiltration was planned, ended up converted to general anaesthesia, had a history of previous abdominal surgery, underwent an emergency (rather than elective) caesarean, or required extra surgical procedures.

A computer-generated schedule assigned participants to one of the three groups in equal proportion. To keep the allocation hidden, an investigator who had no part in recruiting patients, running the anaesthetic, assessing outcomes, or analysing the data prepared sequentially numbered, opaque, sealed envelopes ahead of time.

The study drug for each patient was drawn up right before surgery by an anaesthesiologist who otherwise had nothing to do with the trial. Everyone else involved- patients, the surgical team, the treating anaesthesiologists, nursing staff, the staff assessing outcomes afterwards, and the statistician- stayed blind to group assignment right up until the final analysis was complete. To keep the blinding intact, every study solution was drawn up in syringes that looked identical and held the same total volume regardless of group.

The treatment groups were defined as follows: Group B: 20 mL of 0.25% bupivacaine; Group BDM: 20 mL of 0.25% bupivacaine containing dexmedetomidine (1 μg/kg); Group BDX: 20 mL of 0.25% bupivacaine containing dexamethasone (8 mg).

Patients fasted according to the usual pre-surgical guidance. Once an 18-gauge IV cannula was secured and standard monitors (ECG, non-invasive blood pressure, and pulse oximetry) attached, baseline readings were recorded. Each woman then received Ringer's lactate (10 mL/kg) as a preload ahead of the spinal block. The subarachnoid block was given at either L3-L4 or L4-L5 using a 25-gauge Quincke needle, with 1.8 mL (9 mg) of 0.5% hyperbaric bupivacaine plus fentanyl (10 μg) injected intrathecally. If mean arterial pressure dropped more than 20% below baseline, this was treated as hypotension and corrected with IV fluids plus ephedrine; a heart rate under 50 beats/minute was treated as bradycardia and managed with atropine.

Approximately 30 minutes before the anticipated end of surgery, all patients received 1 g of IV paracetamol infused over 15 minutes. Once the uterus was closed but before skin closure, the operating surgeon infiltrated the allocated study solution along the rectus sheath and wound edges using a sterile 22-gauge needle. After surgery, patients were transferred to the postanaesthesia care unit, where the postoperative protocol below began. Postoperatively, patients received a fixed dose of 1 g IV paracetamol every eight hours. Rescue analgesia followed a weight-based tramadol algorithm: whenever VAS reached 4 or higher, patients received tramadol 0.5 mg/kg IV; if the VAS score had not fallen within a further 10 minutes, an additional dose of tramadol 0.25 mg/kg IV was given, up to a maximum of 1 mg/kg in any six-hour period. A blinded investigator followed a fixed assessment timetable, checking patients first in the post-anaesthesia care unit and later on the ward.

The primary outcome was how much IV tramadol each patient used in total over the first postoperative day. Secondary outcomes included how long analgesia lasted (measured from the moment of wound infiltration to the first rescue dose), pain scores at rest and on movement using a 10-point VAS (0 for no pain, 10 for the worst pain imaginable) recorded at baseline, 15 and 30 minutes, and one, two, six, 12, and 24 hours; a five-point Likert satisfaction rating taken at 24 hours; haemodynamic values through the perioperative period; Ramsay sedation scores [19]; and any adverse events, nausea and vomiting included.

Data were analysed in IBM SPSS Statistics for Windows, Version 25.0 (IBM Corp., Armonk, NY, USA). The Shapiro-Wilk test was applied first to check whether continuous variables followed a normal distribution. Normally distributed variables are summarised as mean ± SD, with between-group comparisons made through one-way ANOVA followed by Bonferroni correction for post hoc testing. For variables departing from a normal distribution, median and interquartile range are reported instead, and group differences were assessed with the Kruskal-Wallis test, followed by Dunn’s procedure for pairwise comparisons where indicated. Categorical data are presented as counts and percentages; these were compared using the chi-square test (χ²), switching to Fisher’s exact test where cell counts were small. Because haemodynamic readings and pain scores were collected repeatedly over time, repeated-measures ANOVA was used to evaluate these serial measurements. All tests were two-tailed, with significance set at P < 0.05.

## Results

Study population and baseline characteristics

Of the women who met the eligibility criteria, 90 were enrolled and split evenly into the three groups: Group B (0.25% bupivacaine), Group BDM (bupivacaine plus dexmedetomidine), and Group BDX (bupivacaine plus dexamethasone). Every enrolled patient finished the trial and went into the final analysis; nobody dropped out, and there were no protocol deviations along the way (Figure 1).

**Figure 1:**
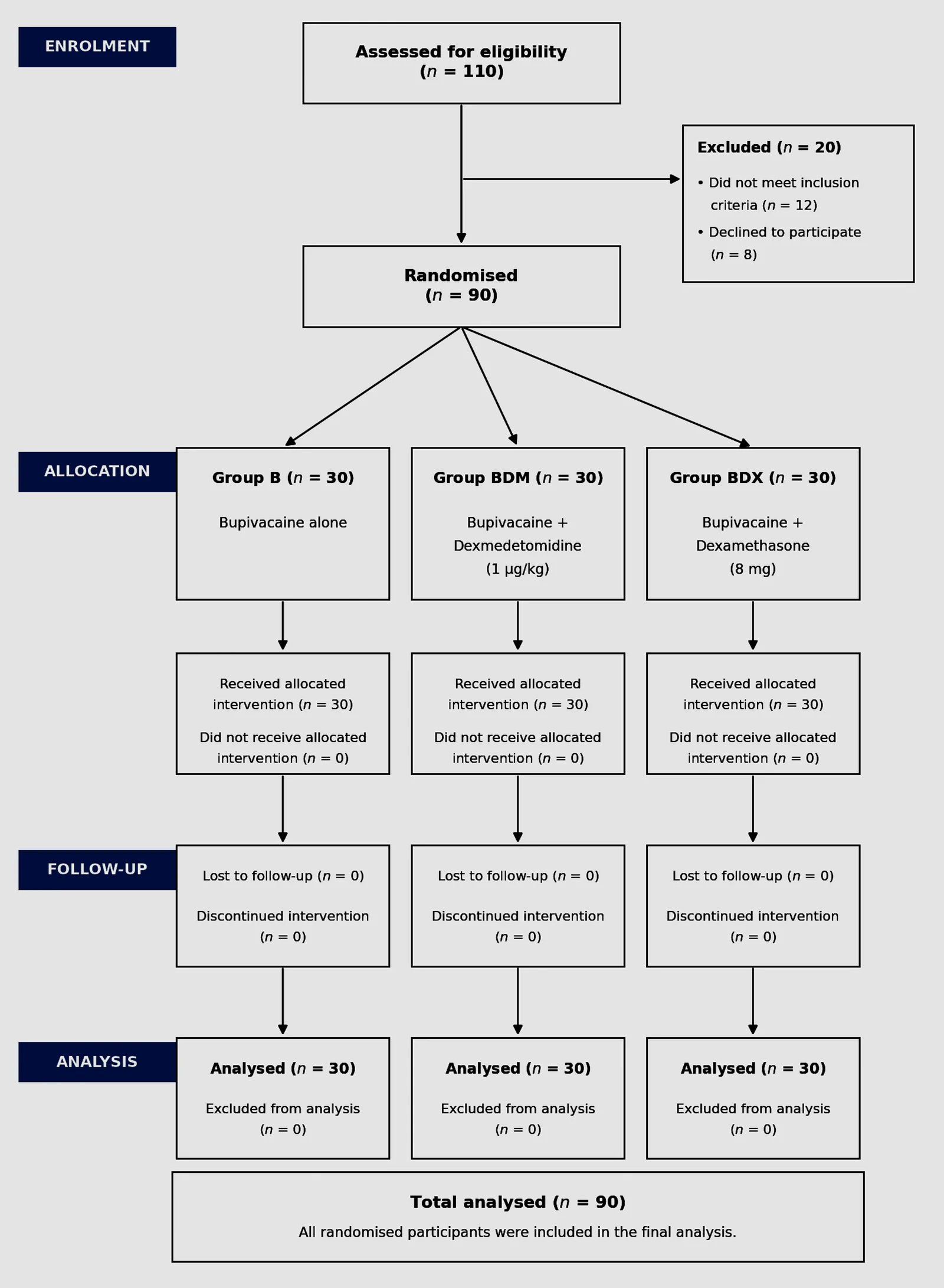
Participant flow throughout the study in accordance with the Consolidated Standards of Reporting Trials (CONSORT) 2010 statement, from screening to final analysis.

Table 1 shows that the three groups looked alike at baseline across the demographic, obstetric, anaesthetic, and perioperative variables measured. Age, weight, height, gestational age, how long surgery took, gravidity, the highest sensory block reached, and the modified Bromage score [20] all came out statistically similar (P > 0.05 throughout), telling us randomisation had done its job, and the groups started on equal footing (Table 1).

**Table 1:** Baseline demographic, obstetric, anaesthetic, and perioperative characteristics. Values are given as mean ± SD for continuous measures and as frequency (%) for categorical ones. Continuous variables were compared across groups with one-way ANOVA (F statistic); categorical variables were compared with the chi-square test (χ² statistic). A two-sided P < 0.05 was taken as the cutoff for significance. — indicates the variable was invariant across groups (100% in all arms) and no test was applicable. Group B: 0.25% bupivacaine; Group BDM: 0.25% bupivacaine plus dexmedetomidine (1 μg/kg); Group BDX: 0.25% bupivacaine plus dexamethasone (8 mg).

Variable	Group B	Group BDM	Group BDX	Test statistic	p-value
Age (years)	25 ± 2.57	25.93 ± 3.05	26.13 ± 3.54	F(2,87) = 1.15	0.319
Weight (kg)	61.8 ± 5.23	60.73 ± 5.45	63.2 ± 5.26	F(2,87) = 1.63	0.203
Height (cm)	157.27 ± 5.03	158.87 ± 4.51	156.7 ± 5.05	F(2,87) = 1.60	0.208
Gestational age (weeks)	37.9 ± 1.12	38.07 ± 1.36	38.67 ± 1.99	F(2,87) = 2.08	0.133
Duration of surgery (min)	46.5 ± 4.58	47.5 ± 5.69	46 ± 5.48	F(2,87) = 0.63	0.535
Primigravida, n (%)	9 (30%)	14 (46.67%)	10 (33.33%)	χ²(2) = 2.01	0.366
Multigravida, n (%)	21 (70%)	16 (53.33%)	20 (66.67%)	χ²(2) = 2.01	0.366
Maximum sensory block level T4, n (%)	30 (100%)	30 (100%)	30 (100%)	—	—
Modified Bromage score Grade 1, n (%)	30 (100%)	30 (100%)	30 (100%)	—	—

Primary outcome

Treatment clearly mattered for how much tramadol women needed over the first 24 hours (P < 0.0001) (Figure 2).

**Figure 2:**
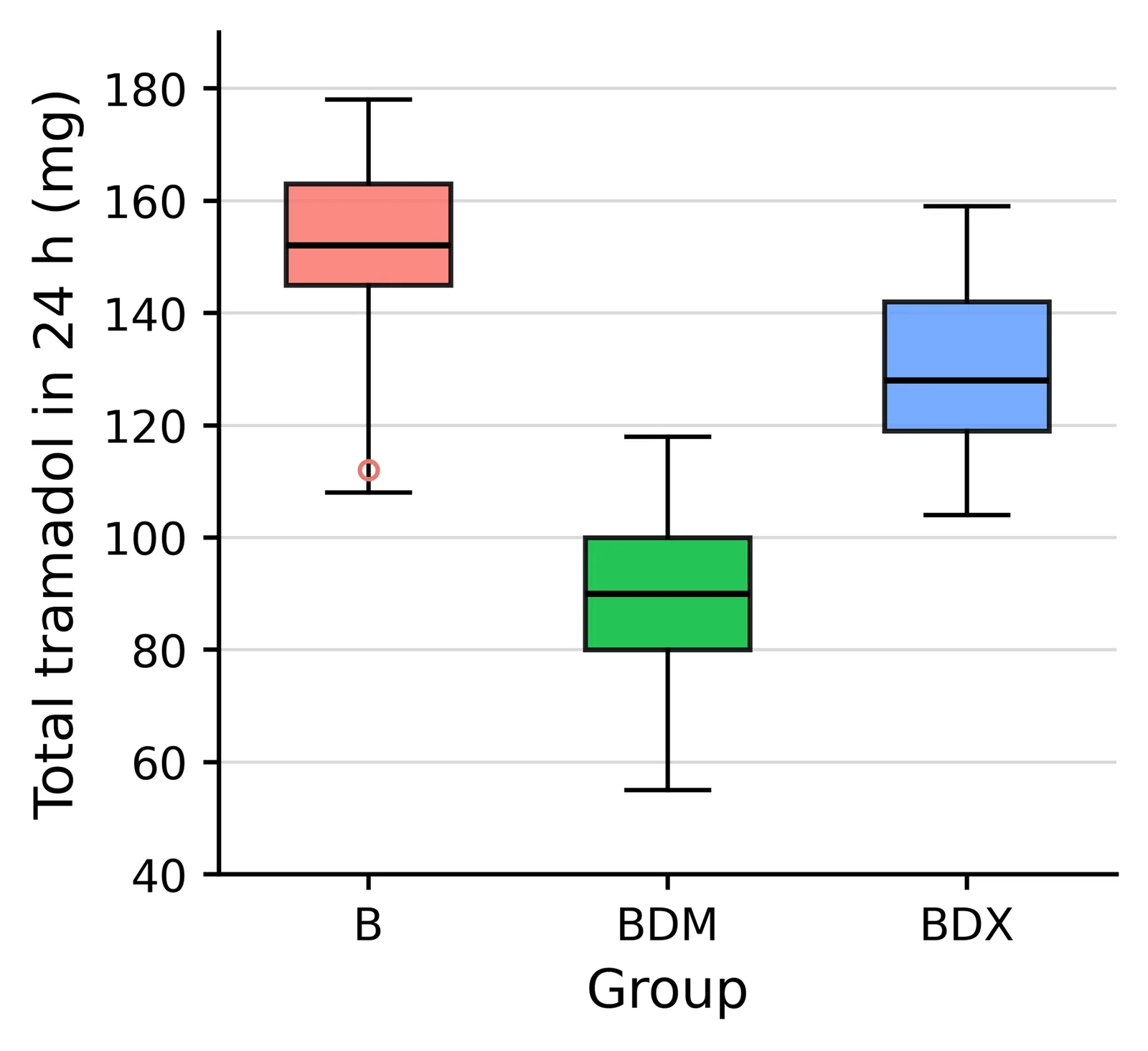
Box-and-whisker plots comparing total tramadol consumption within the first 24 postoperative hours (h) among the three treatment groups. Group B: 0.25% bupivacaine; Group BDM: 0.25% bupivacaine plus dexmedetomidine (1 μg/kg); Group BDX: 0.25% bupivacaine plus dexamethasone (8 mg)

Group BDM required the least 24-hour tramadol consumption (87.33 ± 17.40 mg), Group BDX required more (129.43 ± 15.61 mg), and Group B, on bupivacaine alone, required the most (150.33 ± 14.59 mg). Every pairwise comparison in the post hoc analysis reached significance: Group BDM required less tramadol than both Group BDX and Group B, and Group BDX in turn required less than Group B (all P < 0.0001) (Table 2).

**Table 2:** Postoperative analgesic outcomes and patient satisfaction. Continuous measures are shown as mean ± SD. Group differences were tested with one-way ANOVA (F statistic) followed by Bonferroni-adjusted pairwise post-hoc comparisons (t statistic, df = 87). A P-value below 0.05 was treated as significant. Group B: 0.25% bupivacaine; Group BDM: 0.25% bupivacaine plus dexmedetomidine (1 μg/kg); Group BDX: 0.25% bupivacaine plus dexamethasone (8 mg); PSS: Patient Satisfaction Score; h: hours

Variable	Group B	Group BDM	Group BDX	Test statistic	p-value	t (B-BDM)	p (B-BDM)	t (B-BDX)	p (B-BDX)	t (BDM-BDX)	p (BDM-BDX)
Total tramadol consumption in 24 h (mg)	150.33 ± 14.59	87.33 ± 17.4	129.43 ± 15.61	F(2,87) = 122.05	<0.0001	15.34	<0.0001	5.09	<0.0001	-10.25	<0.0001
Time to first dose of tramadol (h)	1.87 ± 0.35	6 ± 0.10	4 ± 2.03	F(2,87) = 90.26	<0.0001	-13.43	<0.0001	-6.93	<0.0001	6.51	<0.0001
PSS at 24 h (5-point Likert scale)	2.67 ± 0.48	4.5 ± 0.51	3.73 ± 0.45	F(2,87) = 109.64	<0.0001	-14.75	<0.0001	-8.54	<0.0001	6.20	<0.0001

Secondary outcomes

Duration of Analgesia

How long the analgesia held before a rescue dose was needed also varied significantly by group (P < 0.0001): 6.00 ± 0.10 hours in Group BDM, 4.00 ± 2.03 hours in Group BDX, and just 1.87 ± 0.35 hours in Group B (Table 2). Each of these differences held up in pairwise testing (all P < 0.0001)(Figure 3).

**Figure 3:**
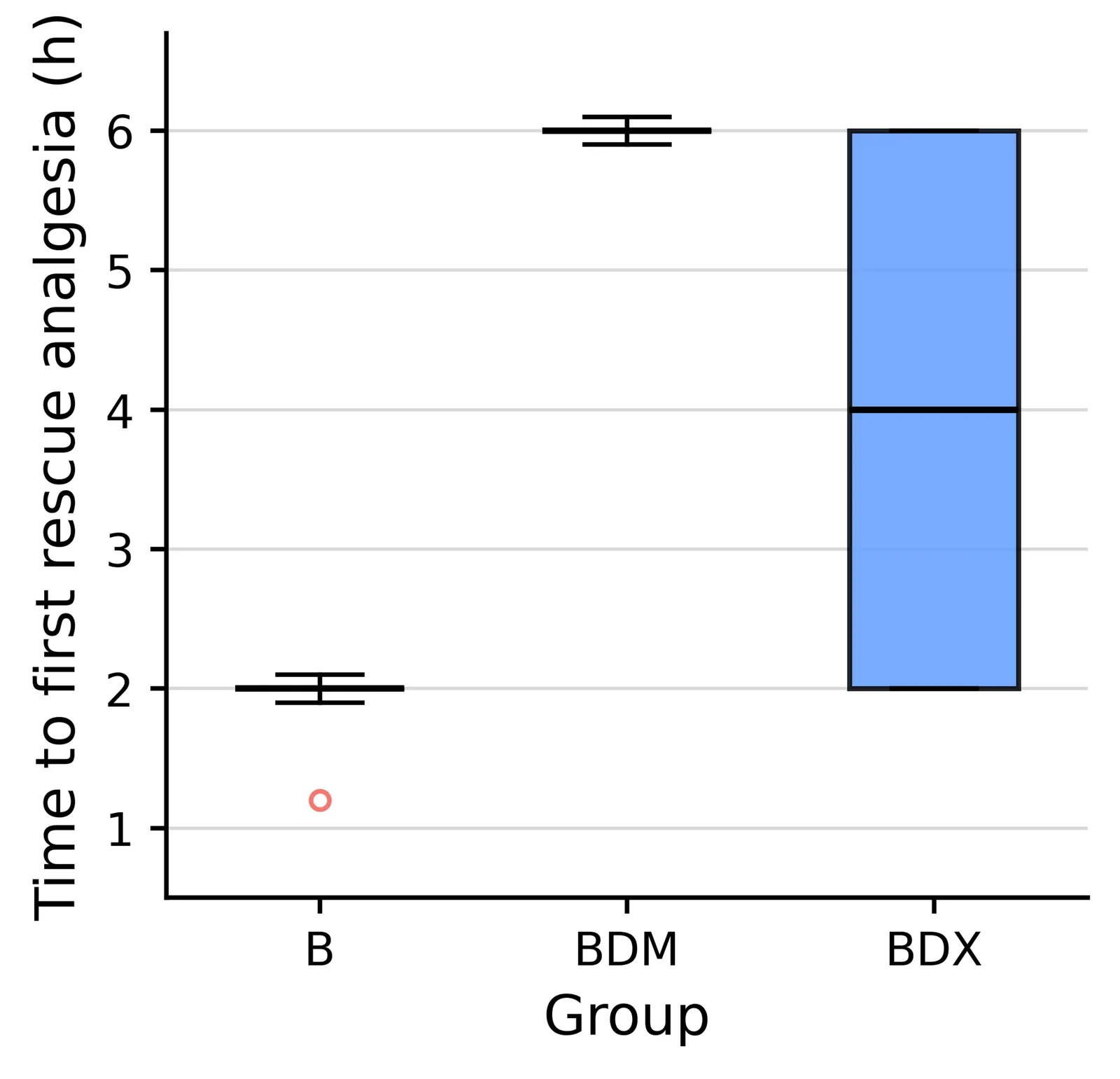
Box-and-whisker plots illustrating the time interval to first rescue analgesia (hours (h)) following surgery across the three study groups. Group B: 0.25% bupivacaine; Group BDM: 0.25% bupivacaine plus dexmedetomidine (1 μg/kg); Group BDX: 0.25% bupivacaine plus dexamethasone (8 mg)

Postoperative Pain Scores

Tracking VAS scores across the full 24-hour window turned up significant between-group differences at nearly every time point (Table 3).

**Table 3:** Postoperative VAS pain scores at rest and during movement over the first 24 hours (h) after surgery. Values are mean ± SD. Overall group differences at each time point were assessed using one-way ANOVA (F statistic), followed by Bonferroni-adjusted pairwise post-hoc comparisons (t statistic, df = 87) where relevant. ‘-’ indicates a comparison not applicable because both groups had zero variance. A P-value below 0.05 was treated as significant. Group B: 0.25% bupivacaine; Group BDM: 0.25% bupivacaine plus dexmedetomidine (1 μg/kg); Group BDX: 0.25% bupivacaine plus dexamethasone (8 mg); VAS: Visual Analogue Scale; min: minutes

Postoperative VAS pain score	Group B	Group BDM	Group BDX	Test statistic	p-value	t (B-BDM)	p (B-BDM)	t (B-BDX)	p (B-BDX)	t (BDM-BDX)	p (BDM-BDX)
At rest											
Baseline (0 h)	0 ± 0	0 ± 0	0 ± 0	F = n/a	1	-	1	-	1	-	1
15 min	0 ± 0	0 ± 0	0 ± 0	F = n/a	1	-	1	-	1	-	1
30 min	1.13 ± 0.35	0 ± 0	0 ± 0	F(2,87) = 312.71	<0.0001	21.66	<0.0001	21.66	<0.0001	0.00	1
1 h	2.13 ± 0.35	0.5 ± 0.51	1.3 ± 0.47	F(2,87) = 99.07	<0.0001	14.08	<0.0001	7.17	<0.0001	-6.91	<0.0001
2 h	2.87 ± 0.35	1.53 ± 0.51	2.5 ± 0.51	F(2,87) = 67.06	<0.0001	11.21	<0.0001	3.10	<0.0001	-8.12	<0.0001
6 h	4.27 ± 0.45	4.7 ± 0.47	3 ± 1.02	F(2,87) = 48.04	<0.0001	-2.38	0.018	7.04	<0.0001	9.43	<0.0001
12 h	5.27 ± 0.45	3.47 ± 0.78	4 ± 1.02	F(2,87) = 41.60	<0.0001	8.87	<0.0001	6.26	<0.0001	-2.61	0.01
24 h	6.27 ± 0.45	5.4 ± 0.81	5.57 ± 0.5	F(2,87) = 17.26	<0.0001	5.54	<0.0001	4.46	<0.0001	-1.08	0.293
During movement											
Baseline (0 h)	0.93 ± 0.25	0 ± 0	0 ± 0	F(2,87) = 415.15	<0.0001	24.95	<0.0001	24.95	<0.0001	0.00	1
15 min	1.03 ± 0.18	0 ± 0	0 ± 0	F(2,87) = 982.31	<0.0001	38.39	<0.0001	38.39	<0.0001	0.00	1
30 min	2.13 ± 0.35	0.63 ± 0.49	1 ± 0	F(2,87) = 151.56	<0.0001	16.71	<0.0001	12.59	<0.0001	-4.12	<0.0001
1 h	3.13 ± 0.35	1.5 ± 0.51	2.3 ± 0.47	F(2,87) = 99.07	<0.0001	14.08	<0.0001	7.17	<0.0001	-6.91	<0.0001
2 h	3.87 ± 0.35	2.5 ± 0.51	3.5 ± 0.51	F(2,87) = 70.34	<0.0001	11.46	<0.0001	3.10	<0.0001	-8.37	0.003
6 h	5.27 ± 0.45	5.67 ± 0.48	4 ± 1.02	F(2,87) = 46.44	<0.0001	-2.21	0.029	7.02	<0.0001	9.23	<0.0001
12 h	6.27 ± 0.45	4.4 ± 0.77	5.5 ± 0.51	F(2,87) = 75.32	<0.0001	12.21	<0.0001	5.03	<0.0001	-7.18	<0.0001
24 h	7.4 ± 0.5	6.53 ± 0.82	6.73 ± 0.64	F(2,87) = 14.03	<0.0001	5.06	<0.0001	3.89	0.0002	-1.16	0.248

Both adjuvants beat bupivacaine alone on pain scores, whether patients were resting or moving, at most of the intervals checked. Dexmedetomidine came out on top most consistently, giving the lowest scores at one, two, 12, and 24 hours, though dexamethasone edged ahead briefly at the six-hour mark (Figure 4).

**Figure 4:**
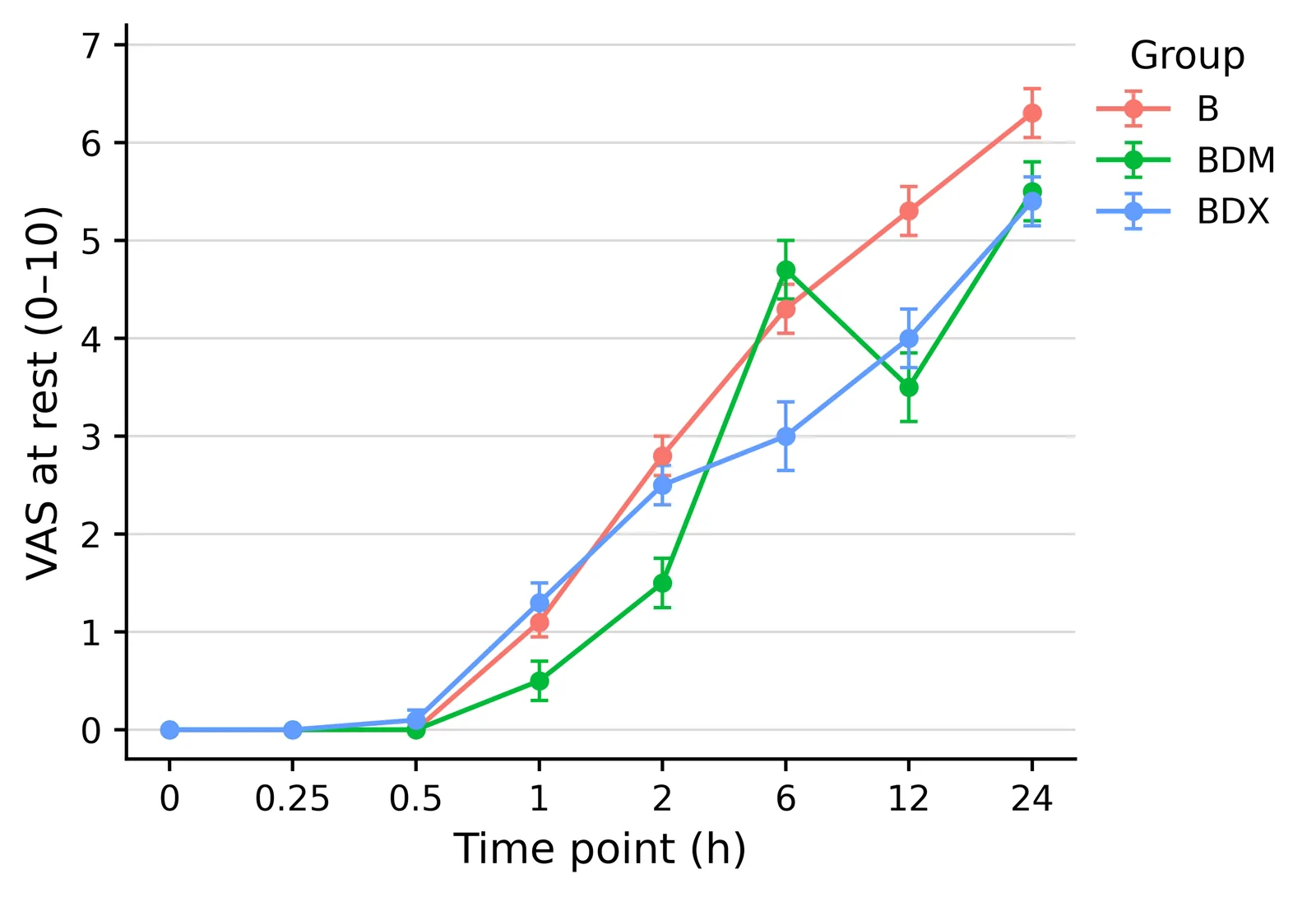
VAS pain scores at rest, tracked at fixed intervals over the 24 hours (h) following caesarean section. Group B: 0.25% bupivacaine; Group BDM: 0.25% bupivacaine plus dexmedetomidine (1 μg/kg); Group BDX: 0.25% bupivacaine plus dexamethasone (8 mg); VAS: Visual Analog Scale

The movement scores followed the same general shape, with both adjuvant arms scoring significantly lower than control at most checkpoints (P < 0.05) (Figure 5).

**Figure 5:**
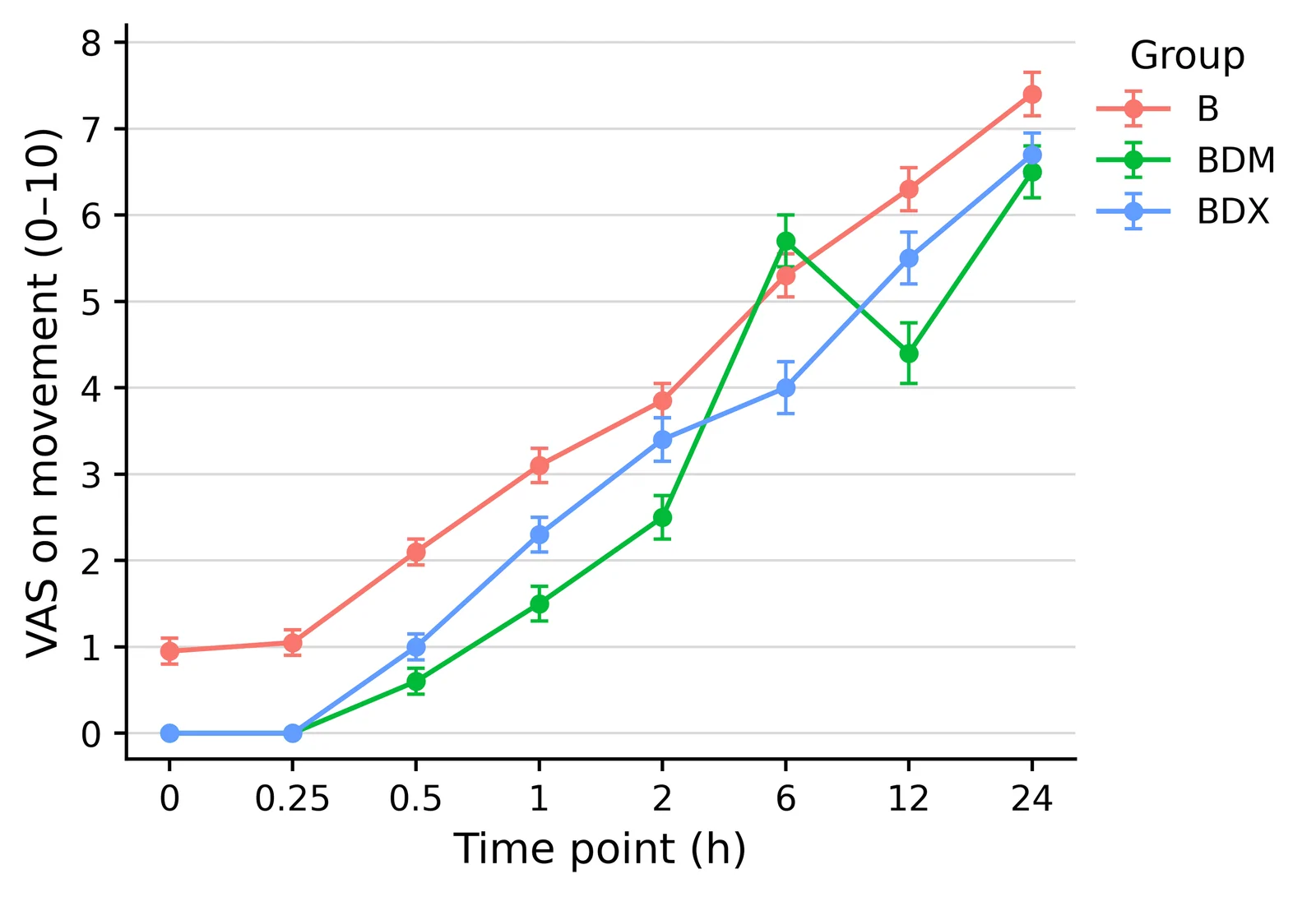
VAS pain scores during movement, tracked at the same fixed intervals over the first postoperative day. Group B: 0.25% bupivacaine; Group BDM: 0.25% bupivacaine plus dexmedetomidine (1 μg/kg); Group BDX: 0.25% bupivacaine plus dexamethasone (8 mg); VAS: Visual Analog Scale; h: hours

Taken together, dexmedetomidine gave the steadiest, longest-lasting pain relief of the three.

Patient Satisfaction

At the 24-hour mark, satisfaction scores also split significantly by group (P < 0.0001)- highest in Group BDM (4.50 ± 0.51), then Group BDX (3.73 ± 0.45), then Group B (2.67 ± 0.48) (Figure 6).

**Figure 6:**
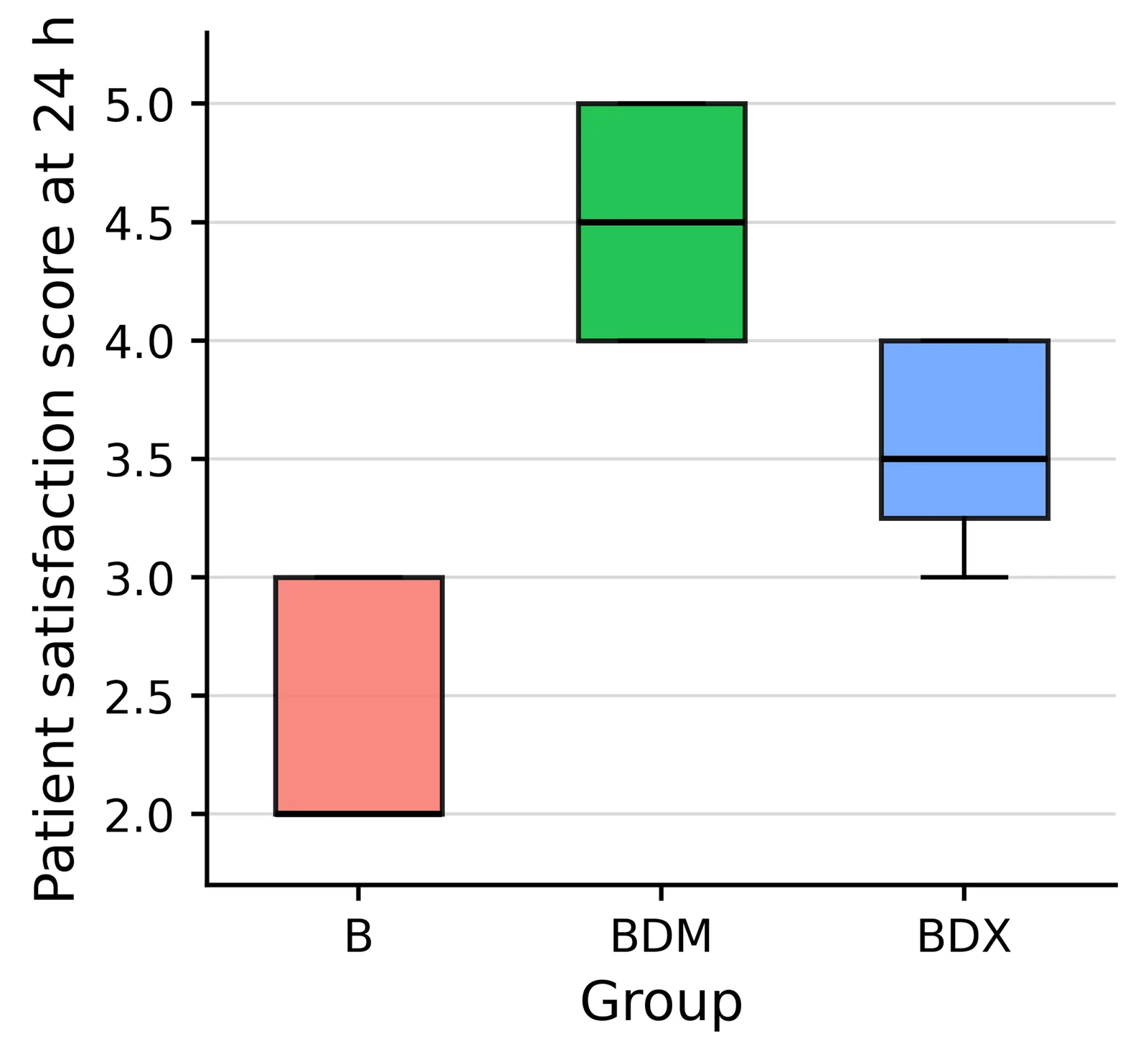
Distribution of maternal patient satisfaction scores at 24 hours (h) after surgery among the three treatment groups, illustrated using box-and-whisker plots. Group B: 0.25% bupivacaine; Group BDM: 0.25% bupivacaine plus dexmedetomidine (1 μg/kg); Group BDX: 0.25% bupivacaine plus dexamethasone (8 mg); VAS: Visual Analog Scale

Post hoc testing confirmed BDM patients were more satisfied than either of the other two groups, and BDX patients in turn were more satisfied than Group B (all P < 0.0001) (Table 2).

Safety outcomes

Heart rate, systolic and diastolic blood pressure, mean arterial pressure, respiratory rate, and oxygen saturation all stayed stable through the perioperative period, with no meaningful group differences at any point checked (all P > 0.05). Group BDM did show mild dips in heart rate and blood pressure afterwards, but these stayed within a clinically unremarkable range and never called for treatment. Respiratory readings and oxygen saturation matched up across all three groups.

Ramsay sedation scores didn’t differ by group at any follow-up point (all P > 0.05), and adverse events overall were uncommon and occurred at comparable rates. Nausea affected four patients (13.3%) in Group B, two (6.7%) in Group BDM, and one (3.3%) in Group BDX (P = 0.338); vomiting occurred in exactly one patient per group (P = 1.000). No cases of serious bradycardia, respiratory depression, allergic reaction, or wound-related complications turned up anywhere in the study (Table 4).

**Table 4:** Postoperative adverse events Categorical variables are presented as numbers (%) and were compared using the chi-square test (χ² statistic). A P-value below 0.05 was treated as significant. Group B: 0.25% bupivacaine; Group BDM: 0.25% bupivacaine plus dexmedetomidine (1 μg/kg); Group BDX: 0.25% bupivacaine plus dexamethasone (8 mg).

Variable	Group B, n (%)	Group BDM, n (%)	Group BDX, n (%)	χ²(2)	p-value
Nausea	4 (13.3%)	2 (6.7%)	1 (3.3%)	2.17	0.338
Vomiting	1 (3.3%)	1 (3.3%)	1 (3.3%)	0.00	1.000

## Discussion

This trial shows that adding either dexmedetomidine or dexamethasone to a bupivacaine wound infiltration sharpens postoperative pain control after an elective caesarean. Both adjuvants helped, but dexmedetomidine came out ahead on every major efficacy measure we tracked. Women given dexmedetomidine used markedly less rescue tramadol, went longer before needing more analgesia, reported lower pain scores, and were more satisfied with how their pain was managed. None of this came at the cost of more adverse events, which points to dexmedetomidine as a genuinely useful addition to multimodal pain regimens after caesarean delivery.

Cutting opioid exposure while still keeping pain under control is one of the central aims of postoperative care, particularly following caesarean delivery, as opioid-related adverse effects may delay early mobilisation, defined as ambulation within the first postoperative day, which is important for reducing the risk of venous thromboembolism, postoperative ileus, and pulmonary complications, and may also adversely affect early maternal-newborn interaction. Here, the dexmedetomidine group used noticeably less tramadol over the first day, which fits with the opioid-sparing role this drug has shown in earlier work. Its analgesic boost is generally attributed to peripheral α₂-adrenoceptor activation that potentiates and extends what the local anaesthetic can do on its own [9,11,14,15]. Dexamethasone also trimmed opioid use relative to bupivacaine alone, but the effect wasn’t as strong as what dexmedetomidine delivered.

Both adjuvants also stretched out how long the pain relief lasted, and the dexmedetomidine group went the longest of all before asking for rescue analgesia, a sign of a more durable effect than dexamethasone managed. Sustained pain relief facilitates early mobilisation, allowing patients to get out of bed and walk sooner after surgery, which lowers thromboembolic and pulmonary risk and supports faster return to normal maternal function, while also improving maternal comfort during the immediate postpartum period. Similar extensions in analgesic duration have been reported in earlier comparable trials [12,13], which lines up with what we found here and backs dexmedetomidine's standing as a useful wound-infiltration adjuvant.

Serial pain assessments demonstrated consistently lower VAS scores in both adjuvant groups than in the control group, with dexmedetomidine providing more sustained analgesia at most postoperative time points. The transient increase in pain scores at approximately six hours in the dexmedetomidine group corresponded to the timing of rescue analgesia rather than loss of treatment effect. These findings are in line with previous randomised studies [12,13] and systematic evidence supporting prolonged analgesia with both dexmedetomidine and dexamethasone [10].

Improved analgesia translated directly into higher satisfaction among women in the dexmedetomidine group. Better pain control, reduced opioid exposure, and greater postoperative comfort probably contributed to these findings, a pattern that echoes previous studies of dexmedetomidine used this way [12,13].

Neither drug raised any real safety concerns. The mild heart-rate and blood-pressure dips seen with dexmedetomidine never reached clinical significance or needed treating, and sedation scores, respiratory measures, and rates of adverse events looked much the same across all three groups. This backs up earlier findings that these adjuvants’ analgesic upside doesn’t come bundled with a higher risk of meaningful complications [9-13].

Put together, these results point to dexmedetomidine-augmented bupivacaine wound infiltration as a practical, effective way to manage pain after an elective caesarean. Given how it cuts opioid use, extends analgesia, lowers pain scores, and boosts satisfaction, all without a safety trade-off, it deserves a place in ERAS-style multimodal protocols.

Strengths

A few design choices here add weight to the findings. Running the trial prospectively, randomised, and double-blind kept selection and observer bias in check, and hiding the allocation plus blinding the outcome assessors added further protection against bias creeping in. Standardising the anaesthetic and perioperative care across groups kept things consistent, and because the groups matched well at baseline, confounding is less of a worry. Looking at several clinically relevant efficacy and safety endpoints together also makes the overall picture more complete and easier to apply in practice.

Limitations

A few caveats apply. Because this was a single-centre trial, how well the results transfer to other settings is uncertain. We also tested just one dose each of dexmedetomidine and dexamethasone, so we can’t say anything about dose-response relationships or whether a different dose might work better. The VAS itself is a subjective measure, and following patients for only 24 hours means we can’t speak to longer-term outcomes. Full blinding wasn’t quite achievable either, since the anaesthesiologist who prepared the solutions necessarily knew which group was which, though that person had no other role in the patient’s care or in assessing outcomes. Larger, multicentre trials that test different dosing regimens over a longer follow-up window would help confirm and build on what we found.

## Conclusions

Adding either dexmedetomidine or dexamethasone to a bupivacaine wound infiltration improved postoperative pain control in women having an elective caesarean under spinal anaesthesia. Of the two, dexmedetomidine came out ahead clinically. It cut opioid use more, kept analgesia going longer, kept pain scores lower, and left mothers more satisfied, all without any safety trade-off. That makes it a strong candidate for opioid-sparing, multimodal analgesic protocols after caesarean delivery, though bigger multicentre trials with longer follow-up are still needed before it’s adopted more broadly.
